# Recovery from Toxic-Induced Demyelination Does Not Require the NG2 Proteoglycan

**DOI:** 10.1371/journal.pone.0163841

**Published:** 2016-10-18

**Authors:** Stefanie Albrecht, Karin Hagemeier, Marc Ehrlich, Claudia Kemming, Jacqueline Trotter, Tanja Kuhlmann

**Affiliations:** 1 Institute of Neuropathology, University Hospital Münster, Münster, Germany; 2 Molecular Cell Biology, Department of Biology, Johannes Gutenberg-University of Mainz, Mainz, Germany; Instituto Cajal-CSIC, SPAIN

## Introduction

NG2 cells are defined as CNS cells expressing chondroitin sulfate proteoglycan nerve/glia antigen. The vast majority of NG2-positive cells also express platelet-derived growth factor receptor alpha (PDGFRα) and are oligodendroglial progenitors (OPC). In addition a subpopulation of pericytes expresses NG2, but is positive for PDGF receptor beta (PDGFRβ) [1]. NG2-positive OPC comprise approximately 5% of the cells in the CNS where they are evenly distributed in grey and white matter [2, 3]. NG2-positive OPC form synapses with neurons [4–6] and react to brain injury with proliferation, as has been shown in several animal models as well as in human demyelinating and degenerative diseases [7–9]. *In vitro*, NG2 positive cells can give rise to oligodendrocytes, astrocytes and occasional neurons depending on cell culture conditions [10–12]. *In vivo*, NG2 cells generate mostly oligodendrocytes as well as small populations of astrocytes as has been demonstrated in fate mapping studies [9, 13–16]. The developmental fate switch from the oligodendroglial into the astrocytic lineage is regulated by Olig2 [17]. A large percentage of NG2 positive cells persists as a self-renewing population in the adult CNS [18–21].

Although NG2 has been extensively used as a marker for OPC, relatively little is known about the functional role of the NG2 proteoglycan. NG2 consists of a small intracellular and a large extracellular domain. The extracellular domain is cleaved by proteases such as ADAM 10 in an activity-dependent fashion, which regulates glutamate signalling at nearby neurons. The NG2 extracellular domain binds receptors, growth factors, extracellular matrix components and proteases (for review see [22, 23]). Lack of NG2 expression in NG2 deficient (NG2-/-) mice or pharmacological inhibition of NG2 ectodomain shedding in wild type OPC results in NMDA and AMPA receptor-dependent reduction of neuronal current amplitudes and an altered behaviour of NG2-/- mice in tests measuring sensorimotor function. These results demonstrate a bidirectional cross-talk between OPC and the surrounding neuronal network [24, 25]. The intracellular domain can be cleaved by the gamma-secretase and may influence the expression of genes, such as prostaglandin D2 synthase which has neuromodulatory properties [26].

NG2 has been reported to promote migration and proliferation in oligodendroglial and neoplastic glioma cells [27–30]. In OPC the effect on migration is mediated via modulation of Rho GTPases and RAC activity and an influence on cell polarity via selective subcellular localization [31, 32]. Contradictory results have been published with respect to the effect of NG2 on de- and remyelination, as well as on inflammation in inflammatory and/or demyelinating animal models. Mice lacking NG2 were reported to show reduced inflammation as well as reduced myelin damage and repair after injection of lysolecithin [33]. In contrast, in EAE experiments using this same NG2-/- mouse line [34] no differences in disease course or extent of de- and remyelination or inflammation was observed [35]. We hypothesized that the effect of a lack of NG2 might be amplified by extended time periods of demyelination. Furthermore, the initial NG2-/- mouse line [34] was generated by insertion of a neo cassette that may affect the function of nearby genes. We thus utilized mice lacking NG2 in which eYFP was inserted in the endogenous NG2 locus [36]. When bred to homozygosity these mice lack expression of NG2. We fed homozygous NG2-/- mice and their wild type littermates (NG2+/+) for 10 weeks with the copper chelator cuprizone which leads to oligodendroglial death and compared the extent of de- and remyelination as well as the degree of inflammation as measured by the numbers of Mac3 (+) microglia/macrophages. In addition, we isolated OPC from NG2-/- as well NG2+/+ mice to compare and analyze oligodendroglial properties prerequisite for remyelination, namely proliferation, migration and differentiation. *In vitro*, NG2-/- OPC demonstrated an increased migratory capacity in PDGF-AA, but not FGF-elicited chemotaxis; however lack of NG2 did not affect proliferation or differentiation of isolated OPC. No effect of NG2 deficiency on de- or remyelination, numbers of myelinated axons, oligodendrocytes or microglia/macrophages was observed.

## Materials and Methods

### Animals

NG2eYFP knock-in mice [24, 37] have been previously described. Heterozygous mice were mated and resulting litters were genotyped using the following primers: NG2eYFP forward 5′-TGACCTTGGATTCTGAGC-3; NG2eYFP-/- reverse 5′-CGCTGAACTTGTGGCCGTTTA-3; NG2eYFP+/+ reverse 5′-ACAGCTTTCCTTCCAGAC-3. Mice lacking or expressing NG2 will be subsequently referred as NG2-/- or NG2+/+, respectively. Animal handling and experiments were conducted according to the German animal protection laws and approved by the responsible governmental authorities (LANUV Nordrhein-Westfalen (AZ 84.-02.04.2012.A427; AZ 8.87–51.05.20.10.262; AZ 8.84.-02.05.20.12.286).

### Cuprizone Model

For de- and remyelination studies, NG2-deficient as well as wild-type littermates (8 weeks of age, body weight between 20 and 25 g, n = at least 5 mice per time point and genotype with normal day/night cycle) were fed with 0.2% (w/w) cuprizone (Sigma Aldrich, C9012) mixed in powdered chow for 10 weeks. Control animals were fed with powdered chow without cuprizone. Mice were sacrificed under deep anesthesia by intracardial perfusion with phosphate-buffered saline (PBS) after 10 weeks of cuprizone diet as well 7 and 14 days after cessation of the diet. Brains were removed and the hemispheres were cut sagittally in the midline. One hemisphere was frozen in liquid nitrogen for RNA analyses, the other hemisphere, together with liver, spleen, and spinal cord was fixed in 4% (w/w) paraformaldehyde (PFA) solved in PBS overnight before embedding it in paraffin.

### Immunohistochemistry

Sections (4 μm) were stained by luxol fast blue periodic acid schiff (LFB-PAS). For this purpose, sections were stained with LFB solution for 72 h, washed with 0.05% LiCO_3_ and subsequently stained with 1% periodic acid followed by Schiff reagent incubation for 20 min. Immunohistochemistry was performed using a biotin-streptavidin peroxidase technique (Dako, K5001) and an automated immunostainer (Autostainer Link 48, Dako). For better antigen retrieval, sections were pretreated with citrate buffer (pH 6) for 40 min. in a steamer. After deparaffinization, intrinsic peroxidase was blocked by incubation with DAKO REAL™ Peroxidase Blocking solution (DAKO, S2023) for 5 min. All antibodies were diluted in DAKO REAL™ Antibody Diluent (DAKO, S0809). Sections were incubated with the primary antibodies rabbit anti-Olig2 (1:150) (IBL, Spring Lake Park, Minnesota, 18953), mouse anti-NogoA (1:10.000) (11c7, a generous gift from M.E. Schwab, Brain Research Institute, University of Zürich and Department of Biology, Swiss Federal Institute of Technology Zürich, Switzerland), mouse anti-Mac3 (1:200) (Mac clone.M3/84, BD Pharmingen, 553322), mouse anti-GFAP (1:4,000) (Dako, Z0334), and mouse anti-Ki67 (1:100) (Abcam, ab16667) for 30 min. at RT. Sections were incubated with secondary biotinylated anti-mouse (1:400) (Amersham Biosciences, Freiburg, Germany), or anti-rabbit (1:400) (Vector Laboratories, Peterborough, UK) antibodies for 15 min. at RT. Nuclei counterstain was performed using DAKO REAL™ Hematoxylin for 5 min. at RT. DAKO REAL™ DAB+Chromogene (DAKO, K3468) was used as color substrate and sections were mounted with Eukitt® mounting medium (O. Kindler GmbH) after dehydration. To determine the extent of de- and remyelination in the caudal corpus callosum LFB-PAS staining was evaluated by a semi quantitative score for myelination (0: ≤ 10%; 1: 11–33%; 2: 34–67% 3: >68% of the area of the dorsal corpus callosum was myelinated). The numbers of cells positive for NogoA, Mac3, or Olig2 were quantified in a blinded fashion in the caudal part of the corpus callosum in at least 7 standardized microscopic fields of 10,000 μm^2^ each which were defined by an ocular morphometric grid.

### Electron Microscopy (EM)

For EM 4 mice from each treatment group were perfused with 10 ml PBS followed by 10 ml Karlsson-Schultz fixation solution (0.1 M phosphate buffer pH 7.4, 0.5% sodium chloride, 4% PFA and 2.5% glutaraldehyde). Brains were removed and incubated for 4 additional days in Karlsson-Schultz fixation solution. The brains were cut coronally and the corpora callosa were dissected (between bregma 1.32 and 0) and embedded in araldite (Agar Scientific, EPOXY RESIN KIT, R1030). Araldite blocks were cut in 1 μm sections, stained with Richardson’s stain (1% methylene blue in 1% borax solution and 1% azur II solution) for examination by light microscopy. Corpus callosum areas were further trimmed and ultrathin sections were prepared. Ultrathin sections were stained with uranyl acetate (Serva, 77870) as well as Reynold’s lead citrate and examined by transmission electron microscopy. The quantification of myelinated axons were done in at least 4 different regions including only axons with a diameter of more than 0.30 μm. Additionally, we quantified the g-ratio (axon diameter/fibre diameter) of least 100 axons (> 0.30 μm in diameter) per animal using the NIH ImageJ software.

### Generation of primary murine oligodendrocyte precursor cells (OPCs)

OPCs were isolated by sequential immunopanning and kept under proliferative conditions as described earlier [38] until the onset of experiments. Briefly, the brains of postnatal day 6–9 NG2-/- deficient or NG2 wild type mouse brains were dissected, chopped, and triturated. The obtained single cell suspension was resuspended in panning buffer and transferred to a bacterial culture dish coated with Anti-BSL1 Griffonia simplicifolia lectin (Vector Labs, L-1100) for negative selection, followed by a positive selection step using rat anti-mouse CD140a (Biolegend, 135902) as primary antibody and AffiniPure goat anti-rat IgG (H + L) (Dianova, 112-005-003) as secondary antibody enabling a purity of 95% OPC. The adherent OPCs were washed with PBS and detached in OPC culture medium [38] using cell scrapers and plated in culture flasks coated with poly-L-lysine (PLL). The OPCs were cultured in the presence of platelet-derived growth factor-AA (PDGF-AA) (10 ng/ml) (Peprotech, 100-13A) and NT3 (5 ng/ml) (Peprotech, 450–03). To induce differentiation PDGF-AA was replaced by ciliary neurotrophic factor (CNTF) (10 ng/ml) (Peprotech, 450–13).

### Cell Based Assays—viability, proliferation, migration, and differentiation

*Viability* of oligodendroglial cells was determined under proliferating and differentiating conditions according to manufacturer’s protocol using CellTiter-Glo^®^ Luminescent Cell Viability Assay (Promega, G7570). Cell *proliferation* was determined measuring Bromodeoxyuridine (BrdU) incorporation (Cell Proliferation ELISA, BrdU (colorimetric), Roche Diagnostics, 11647229001). Cell viability and proliferation was measured with the GloMax®-Multi Detection System (Promega).

OPC *migration* (chemokinesis) was analyzed using the JuLI™ Br Live Cell Analyzer (Peqlab) for chemokinesis experiments. Using the JuLI™ Live Cell Analyzer NG2-/- and NG2+/+ OPCs were plated in culture medium with either PDGF-AA (30 ng/ml) or FGF2 (20 ng/ml)(R&D Systems). Images were taken simultaneously every 15 min. for 18 hours and the generated movies were analyzed for total cell movement per μm as well as to their average speed in μm per min. The analysis was performed using the MTrackJ plugin for ImageJ (NIH systems). Chemotaxis was determined via impedance measurements using the xCELLigence system. Cells were plated in the PLL-coated upper chamber of a CIM-Plate16 (ACEA Biosciences). To stimulate OPC migration, 30 ng/ml PDGF-AA was added in the culture medium of the lower chamber. The impedance was measured every 15 min. for 24 h and migration was quantified according to manufacturer’s protocol (xCELLigence, RTCA DP Analyzer, RTCA software 1.2, ACEA Biosciences).

Oligodendroglial *differentiation* was assessed by three different assays: cell morphology, immunocytochemistry (ICC) and quantitative RT-PCR (qRT-PCR). For the evaluation of cell morphology, oligodendroglial differentiation was induced and images were taken after 6, 24, 30 and 48 h. Cell processes of 100 cells per time point were counted and classified as oligodendroglial progenitor (0–2 processes), immature (3–13 processes) or mature (differentiated cells with myelin sheet formation) oligodendrocytes. For ICC, OPCs were differentiated for 48 hours and fixed in 4% PFA for 20 min. at RT. Cells were permeabilized for 10 min. in 0.5% Triton X-100 in PBS and unspecific antibody binding was blocked using 5% FCS (v/v) in PBS for 30 min. The primary antibodies were rat anti-MBP (1:200) (Abcam, Ab7349) and rabbit anti-PDGFRα (1:300) (Santa Cruz, SSC338). Incubation was performed at 4°C over night. Secondary antibody staining was performed using Cy™3 AffiniPure Goat Anti-Rat IgG (H+L) (1:500) (Jackson, 112-165-167) and donkey anti-Rabbit IgG (H+L), Alexa Fluor® 647 conjugate (1:500) (Invitrogen, A31573) for 2 hours at RT before embedding it in Roti®-Mount FluorCare DAPI (Carl Roth, HP20.1). Images were taken using the laser scanning microscope (LSM) 700 (Zeiss Jena) and the Imager M2 (Zeiss Jena). At least 200 cells were quantified and the numbers of MBP(+) and PDGFRα(+) were assessed as percentage of total DAPI(+) cells. All experiments were repeated at least three times.

### RNA isolation and quantitative Real-Time PCR

Total RNA from cells or corpus callosum was isolated using peqGOLD Total RNA Kit, (PeqLab Biotechnologie GmbH, 12–6634) according to manufacturer's protocol. Quantification of total RNA was performed with Nanodrop ND1000 (Peqlab). mRNA was transcribed into cDNA using the High Capacity cDNA Transcription Kit (Applied Biosystems, 4368813). cDNAs were diluted to a final concentration of 0.75 ng/μl. All qRT-PCRs were carried out using the StepOne Plus real time cycler (Applied Biosystems) and the KAPA SYBR FAST ABI Prism master mix (Peqlab, 07-KK4603-03). The melting curve of each sample was determined to ensure the specificity of the products. The following primers were used: *Pdgfra* forward 5′-CTGCCAGCTCTTATTACCCTCT-3′; *Pdgfra* reverse 5′-TTAGCTAGCGGCCGCGC AGCACATTCATACTCTCCAC-3′; *Mbp* forward 5′-AAGAACATTGTGACACCTCGAA-3′; *Mbp* reverse 5′-CTCTTCCTCCCAGCTTAAAGAT-3′; *Plp1* forward 5′-CAAGACCTCTGCCAGTATAG-3′; *Plp1* reverse 5′-AGATCAGAACTTGGTGCCTC-3′; *Mag* forward 5′-ACCGCCTTCAACCTGTCTGT-3′; *Mag* reverse 5′-CTCGTTCACAGTCACGTTGC-3′; *Il-1ß* forward 5′-GCCCATCCTCTGTGACTCAT-3′; reverse 5′-AGGCCACAGGTATTTTGTCG-3′; *Ifng* forward 5′-CAAGTTTGAGGTCAACAACCCACA-3′; *Ifng* reverse 5′-CCACCCCGAATCAGCAGCGAC-3′ All results were normalized to the housekeeping gene *RPLP0*. *RPLP0* forward 5′-CGACCTGGAAGTCCAACTAC-3′, *RPLP0* reverse 5′-ATCTGCTGCATCTGCTTG-3′. Cycling conditions consisted of an initial heating period over 10 min. at 95°C, followed by 40 cycles; each cycle consisted of denaturation at 95°C for 15 sec, annealing for 15 sec, and extension at 72°C for 1 min. All samples were processed as technical triplicates and were analyzed by the Pfaffl ΔΔCt method.

### Statistics

All cell culture experiments were performed in triplicates and replicated at least three times if not mentioned otherwise. In text and figures, the results are provided as mean ± SEM. Student-t test or Bonferroni-corrected one- or two-way ANOVA tests (multiple comparisons) were performed for statistical analysis. Tests were considered significant with *p* values < 0.05. All statistical analyses were performed using GraphPad Prism 5.03 (GraphPad Software, San Diego, CA).

## Results

### NG2-/- OPC demonstrate an increased PDGF-AA elected chemotaxis, but no differences in chemokinesis, viability, or proliferation

OPC were isolated from P 6–9 NG2-/- or NG2+/+ mouse brains using a sequential immunopanning protocol resulting in a purity of >95% [38]. Culturing of cells in the presence of PDGF-AA and NT3 maintains them in a proliferative und migratory stage; replacing PDGF-AA by CNTF results in the differentiation into mature oligodendrocytes.

**Fig 1:**
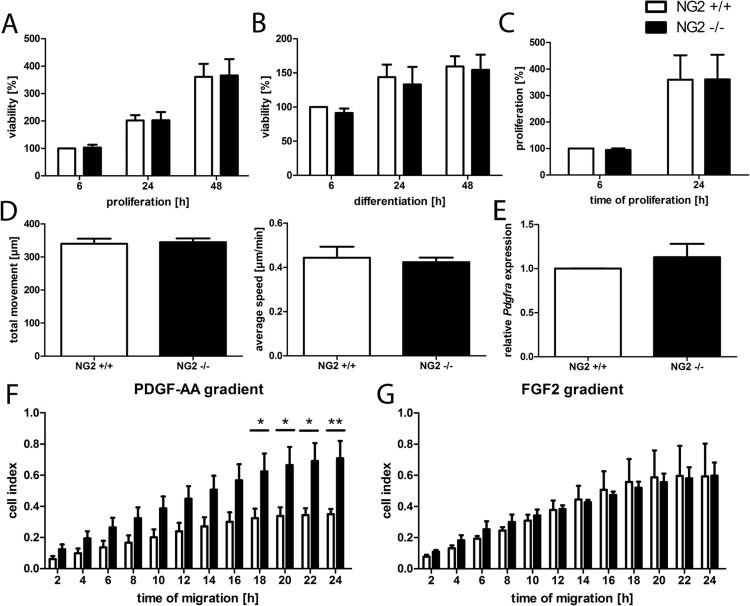
PDGF-AA elicited increased directed migration (= chemotaxis) in NG2-/- OPCs. Viability was comparable between NG2-/- and NG2+/+ oligodendroglial lineage cells under proliferating (A) or under differentiating (B) conditions. Additionally, no differences could be observed comparing the proliferative activity of NG2-/- and NG2+/+ OPCs after 24 h (n = 4) (C). No differences in total movement or average speed between NG2-/- and NG2+/+ were observed in chemokinesis assays. Using PDGF-AA as chemoattractant significantly increased chemotaxis of NG2-/- compared to NG2+/+ OPCs was observed (n = 5) (F). When FGF2 was used as chemoattractant, chemotaxis was comparable between the two genotypes (n = 4) (G). NG2-/- and NG2+/+ OPCs express comparable levels of *Pdgfra* mRNA (n = 5) (E).

Viability was determined after 6, 24 and 48 hours under proliferating and differentiating conditions. No differences in cell viability were observed between the two genotypes (Fig 1A and 1B). To analyze proliferation, BrdU assays were performed. No difference was detected comparing NG2-/- and NG2+/+ OPCs (Fig 1C). Additionally, we determined the migratory capacity of NG2-/- and NG2+/+ OPCs analyzing undirected (chemokinesis) as well as directed (chemotaxis) migration. To examine chemokinesis, live cell imaging and cell tracking was performed. No differences in total movement or the average speed could be determined (Fig 1D, S1 and S2 Movies). In contrast, NG2-/- OPC demonstrated a significant higher directed migration compared to NG2+/+ OPC if PDGF-AA was used as a chemoattractant (Fig 1F). However, this difference was not observed if FGF2 was used as chemoattractant instead of PDGF-AA (Fig 1G). To determine whether different expression patterns of the receptor PDGFRα may contribute to the differences observed in PDGF-AA elicited chemotaxis, we analyzed the expression levels of PDGFRα in NG2+/+ and NG2-/- OPCs. No difference in *Pdgfra* was observed (Fig 1E).

### Loss of NG2 does not affect oligodendroglial differentiation

**Fig 2:**
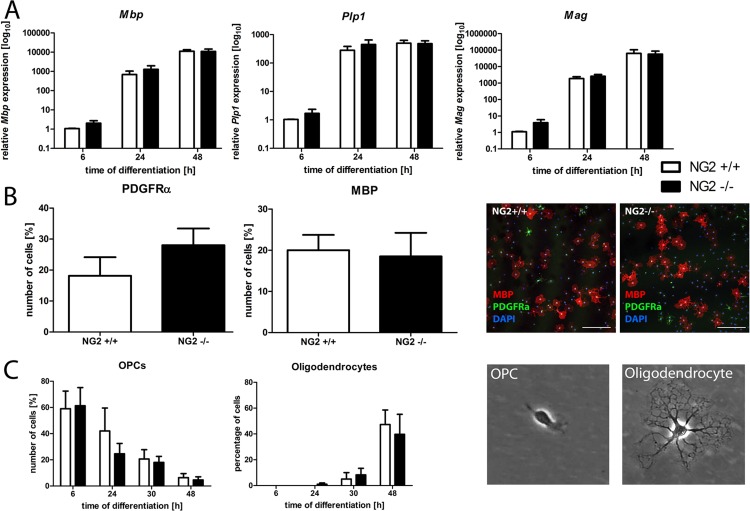
Loss of NG2 does not affect oligodendroglial differentiation. Using qRT-PCR no differences in the relative expression of the myelin associated genes *Mbp*, *Plp1*, or *Mag* were observed comparing NG2-/- and NG2+/+ cells (n = 5) (A). Immunocytochemistry revealed no differences in the number of PDGFRα(+) and MBP(+) cells after 48 h of differentiation (n = 6). Representative pictures of differentiated cultures are shown (B). Also the evaluation of cell morphology demonstrated comparable differentiation of the two genotypes differentiation after 6, 24, 30, or 48 h (C). Scale bars represent 200 μm.

For the evaluation of oligodendroglial differentiation OPC were allowed to differentiate into mature oligodendrocytes over 48 hours. Samples were taken after 6, 24, and 48 hours. The mRNA expression levels of the myelin associated genes *Mbp*, *Plp1*, and *Mag* increased over time, but no differences in the relative expression levels between NG2-/- and NG2+/+ oligodendrocytes were detected (Fig 2A). Furthermore, we quantified the number of processes of NG2-/- and NG2+/+ oligodendrocytes 6, 24, 30, and 48 hours after initiation of oligodendroglial differentiation. As expected the number of processes increased during oligodendroglial differentiation; however no differences between the two genotypes were observed (Fig 2C). Using immunocytochemistry we determined the number of MBP-positive mature oligodendrocytes and PDGFRα-positive OPC after 48 hours. Again, no differences were detected (Fig 2B).

### Lack of NG2 had no effect on remyelination after cuprizone-induced demyelination

**Fig 3:**
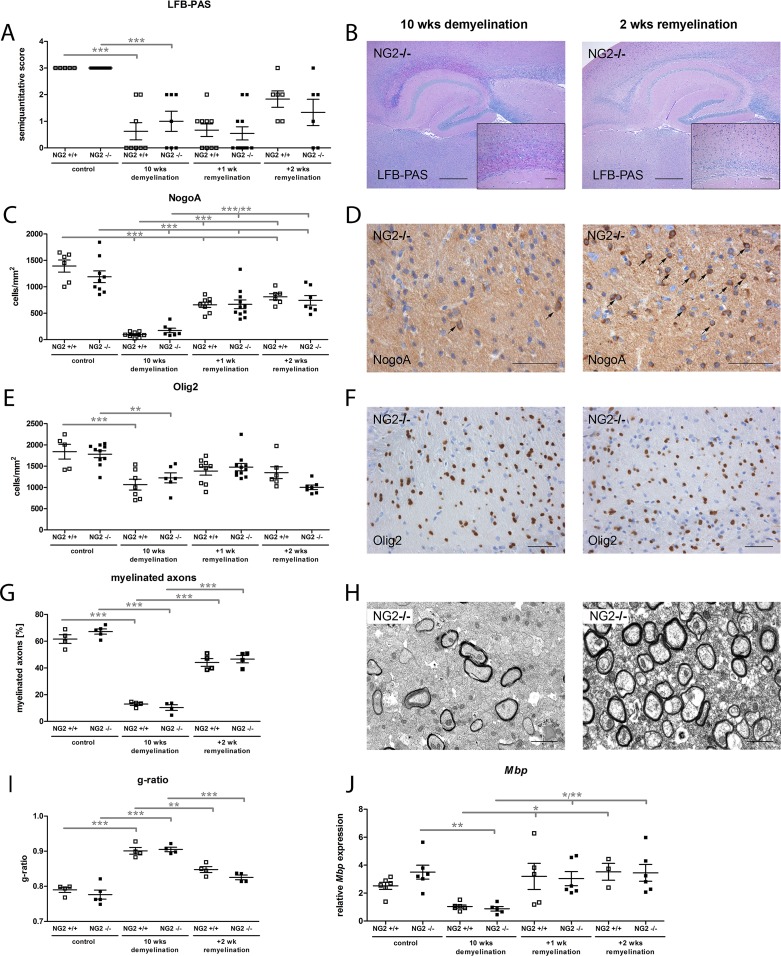
NG2 is dispensable for successful remyelination in the cuprizone model. After 10 weeks of cuprizone induced demyelination and during subsequent remyelination (7 and 14 days after cessation of cuprizone diet) no differences in the extent of myelination (LFB-PAS) (A-B), the numbers of NogoA(+) mature oligodendrocytes (C-D) or Olig2(+) cells (E-F) were detected between NG2-/- and NG2+/+ mice. Furthermore, the quantification of myelinated axons and the g-ratio by EM revealed no differences between both genotypes (G-I). The expression level of *Mbp* was similar in NG2-/- and NG2+/+ mice after 10 weeks of demyelination and 1 and 2 weeks of remyelination (J). Scale bars represent 500 μm and 100 μm respectively (B & insert), 50 μm (D, F), and 1 μm (H).

**Fig 4:**
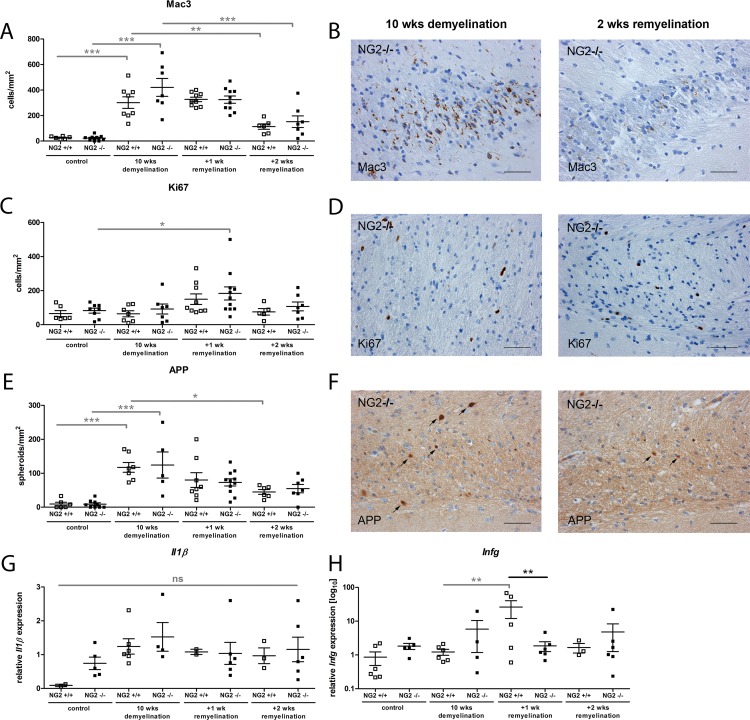
No difference in the numbers of microglia/macrophages, proliferating cells and axonal damage between NG-/- and NG2+/+ mice in the cuprizone model. After 10 weeks of demyelination and during subsequent remyelination (7 and 14 days after cessation of cuprizone diet) no differences in the numbers of microglia/macrophages (Mac3) (A-B) or proliferating cells (Ki67+) (C-D) were observed. Furthermore, the extent of axonal damage measured by APP-positive spheroids was comparable in NG2-/- and NG2+/+ mice during de- and remyelination (E-F). qRT-PCR revealed no differences in the expression levels of *Il1ß* between NG2-/- and NG2+/+ mice (G) and the expression of *Infg* was reduced in NG2-/- mice after 1 week of remyelination (H). Scale bars represent 50 μm (B, D, and F).

In order to evaluate the functional role of NG2 during de- and remyelination, we used the cuprizone model. NG2-/- and NG2+/+ mice were fed for 10 weeks with cuprizone [39, 40]; as expected this resulted in extensive demyelination especially in the dorsal part of the corpus callosum as demonstrated by reduced LFB-PAS staining and significantly lower numbers of NogoA (expressed by mature oligodendrocytes) or Olig2 (expressed by OPC and mature oligodendrocytes) positive oligodendroglial lineage cells (Fig 3A–3F). Demyelination was further confirmed by EM demonstrating reduced numbers of myelinated axons and an increased g-ratio (axon diameter/fibre diameter) (Fig 3G–3I); additionally, reduced *Mbp* mRNA levels were detected (Fig 3J). Demyelination was associated with an increase in Mac3 positive macrophages/microglia in both NG2-/- and NG2+/+ mice (Fig 4A and 4B) in the corpus callosum. After cessation of cuprizone feeding, spontaneous remyelination occurred that was assessed after 7 and 14 days. During remyelination no difference between the two genotypes was observed. Similarly, in NG2-/- and NG2+/+ mice comparable numbers of myelinated axons, g-ratios and Olig2 or NogoA positive oligodendrocytes as well as *Mbp* levels were found using EM, immunohistochemistry and quantitative RT-PCR analyzes after 7 and 14 days of remyelination (Fig 3). Interestingly, even after two weeks of remyelination the number of mature NogoA positive oligodendrocytes was still significantly reduced compared to untreated control mice. We observed a slightly increased number of proliferating Ki67 positive cells after 1 week of remyelination compared to mice without cuprizone feeding; this increase was significant for NG2-/- but not NG2+/+ mice (Fig 4C and 4D). However, no significant difference between both genotypes was observed. To analyze axonal damage, we stained for amyloid precursor protein (APP) that accumulates in injured axons at the site of disturbed axonal transport. APP-positive axonal spheroids may persist for weeks or months [41] and the axons in which APP accumulates either degenerate or recover [42]. We observed a slight increase in the number of APP-positive axonal spheroids after 10 weeks of demyelination followed by a slow decrease during remyelination; but no differences between the genotypes were observed (Fig 4E and 4F). Since recent data demonstrated that loss of NG2 lead to downregulation of pro-inflammatory cytokines in a focal demyelinating model [33] we analyzed the expression levels of *Il1β* and *Infg* in the NG2-/- and NG2+/+ mice after de- and remyelination. We detected a slightly decreased *Infg* expression in NG2-/- animals after 1 week of remyelination; no further differences in *Ifng* or *Il1β* expression between the two genotypes were observed.

## Discussion

NG2-positive OPC are responsible for developmental myelination well as remyelination after a demyelinating event. Although NG2 is a widely used marker to identify OPC, relatively little is known about the functional role of NG2 expressed by oligodendroglial lineage cells. The aim of our study was to investigate the functional role of NG2 for oligodendroglial proliferation, migration, differentiation and remyelination in a demyelinating model in which mice are fed cuprizone.

For our *in vitro* and *in vivo* experiments we used mice in which the endogenous NG2 is replaced by EYFP. In contrast to earlier NG2-/- mouse lines the neo cassette which may affect the function of nearby genes was removed [36]. Using isolated NG2-/- and NG2+/+ oligodendrocytes we observed an increased PDGF-AA elicited chemotaxis, but no differences in oligodendroglial viability, proliferation or differentiation between NG2-/- and NG2+/+ mice. In the cuprizone model, no differences between NG2-/- and NG2+/+ mice were observed with respect to de- and remyelination, inflammation, axonal damage or oligodendroglial proliferation and differentiation.

Migration of OPC is regulated by a large number of environmental cues, among them growth factors such as PDFG-AA and FGF2 (for review see [43], [44–47]. The exact pathways by which PDGF activates migration of OPCs is unknown; however activation of Cdk5 by Fyn followed by phosphorylation of WAVE2, activation of the ERK pathway, increase of intracellular Ca^**2+**^ levels and modulation of components of the cytoskeleton may be involved [44, 48–50]. The downstream signalling pathways modulating oligodendroglial migration induced by FGF2 are less well studied. FGF2 mediates its effect on migration via FGFR1, one of 4 FGF receptors, most likely in a PDGF-AA-independent mechanism [45, 51, 52]. So far only one study examined the functional role of NG2 for oligodendroglial migration. Oli-neu cells stably transfected with control or shRNA directed against NG2 demonstrated decreased directed migration in response to FGF2, potentially via activation of the RhoA/Rock pathway [31, 32]. Similarly, we observed an effect of NG2-deficiency on directed migration; however, in our experiments lack of NG2 resulted in increased directed migration in response to PDGF-AA, but not FGF2. The differences in the results might be explained by the different cell populations used (primary murine oligodendroglial cells vs. Oli-neu cells). However, despite the discrepancies, our results support the notion that FGF2 and PDGF-AA modulate oligodendroglial migration via different signalling cascades [45]. Migration of oligodendrocytes may be also regulated by NMDA receptors [53]. Interestingly, neuronal NMDA receptor dependent long term potentiation and AMPA receptor subunit properties are changed in NG2-/- compared to wildtype mice [24]. However, there is no indication that NG2-/- OPC have altered NMDA receptor composition; furthermore the glutamatergic synapses appear normal in NG2-/- OPC [54]. The signaling cascades *in vivo* regulating oligodendroglial proliferation are not well understood. Earlier publications have demonstrated a reduced proliferation and delayed differentiation of OPC in NG2-deficient mice during developmental myelination or after lysolecithin-induced demyelination [27, 33]. Additionally, NG2-deficient keratinocytes or glioma cells demonstrated reduced proliferation [30, 55–57]. Using isolated OPC from NG2-/- and NG2+/+ mice we did not observe a difference in oligodendroglial proliferation or differentiation *in vitro*. However, the above mentioned publications demonstrated decreased oligodendroglial proliferation and delayed oligodendroglial differentiation in NG2-/- mice in *in vivo* studies, a more complex situation. In the area of a stab wound differences in the accumulation and polarity of OPC were seen between NG2-/- and NG2+/+ mice [31]. In a model of traumatic brain injury (TBI) where a controlled cortical impact was used, strikingly worse clinical outcome was seen in mice lacking NG2 compared to WT littermates [58]. An up-regulation of the chemokine CXCL13 in macrophages, enhanced infiltration of CD45+ leukocytes and an increase of activated Iba-1+/CD45+ microglia/macrophages was observed in NG2-/- mice 30 days after TBI. In both these models where striking differences between NG2-/- and NG2+/+ mice were seen, there is an extensive inflammation with blood brain barrier damage and it is conceivable that the NG2 proteoglycan itself, possibly as a shed ectodomain, modulates the inflammatory response. Since it has been shown that cleavage of the extracellular NG2 domain regulates neuronal networks [24] it might be also possible that neuronal signaling in turn modulates oligodendroglial properties, such as proliferation or differentiation. To further study potentially indirect effects of NG2 on oligodendroglial proliferation and differentiation *in vivo* we used the cuprizone model. Cuprizone is a copper chelator that induces oligodendroglial cell death followed by demyelination, astrogliosis and microglia activation. Since the blood brain barrier is not affected only few blood derived monocytes and T cells invade the CNS; furthermore axonal damage is less pronounced that in inflammatory demyelinating models such as EAE [59, 60]. OPC are already recruited during ongoing demyelination, but the formation of new myelin sheaths starts after cessation of the cuprizone diet [61]. Using Olig2 as a marker that labels OPC as well as mature oligodendrocytes, we observed a significant increase in the number of total oligodendrocytes during remyelination, but there was no difference in the number of Olig2-positive or Ki67 positive proliferating cells between NG2-/- and NG2+/+ mice, suggesting that NG2 has no major effect on oligodendroglial proliferation *in vivo* in this model. Similarly, in NG2-/- and NG2+/+ mice comparable numbers of mature NogoA-positive were found indicating that NG2 has also no major effect on oligodendroglial differentiation, at least not in the cuprizone model. However, we cannot exclude that the observed differences between the different *in vivo* studies might be either due to the different animal models and a variable immune cell/OPC interactions or different NG2 knock-out mice used.

Newly differentiated oligodendrocytes derived from adult OPC are responsible for remyelination [62–64]. Earlier studies reported delayed de- and remyelination in lysolecithin-induced demyelinating lesions; this was associated with an increased expression of anti- and a downregulation of pro-inflammatory cytokines [33]. The decrease in remyelination was associated with reduced proliferation of progenitors; whether delayed oligodendroglial differentiation might have contributed to delayed remyelination was not assessed. In contrast, using the same NG2-/- mouse line no effect on inflammation, extent of demyelination or remyelination was observed in EAE [35]. The differences in remyelination between the two studies might be explained by the different animal models; however the assessment of remyelination in EAE is difficult since the age of the lesion and the time point of initiation of remyelination is not known for an individual lesion. In the cuprizone model we observed comparable numbers of proliferating cells, numbers of Olig2 or NogoA positive oligodendrocytes as well as myelinated axons between NG2-/- and NG2+/+ mice, suggesting that oligodendroglial proliferation and differentiation as well as remyelination were not affected by NG2 deficiency. These findings suggest that NG2 is dispensable for successful remyelination after cuprizone-induced demyelination

In summary, we have shown here that lack of NG2 affects directed migration of isolated oligodendrocytes; but has no effects on proliferation, differentiation or remyelination *in vivo* in a model where little adaptive inflammation is induced. To elucidate the exact mechanisms how NG2 regulates oligodendroglial properties such as migration further studies are required. In particular future work should focus on the interplay between NG2-expressing cells and the immune system in these complex *in vivo* situations where inflammation is a striking component.

## Supporting Information

S1 Movie Movie of NG2 +/+ OPCs in chemokinesis assay over 18h. (MPG)

S2 Movie Movie of NG2 -/- OPCs in chemokinesis assay over 18h. (MPG)

## References

[1] Murfee WL, Skalak TC, Peirce SM. Differential arterial/venous expression of NG2 proteoglycan in perivascular cells along microvessels: identifying a venule-specific phenotype. Microcirculation. 2005;12(2):151–60. 15824037 10.1080/10739680590904955

[2] Nishiyama A, Boshans L, Goncalves CM, Wegrzyn J, Patel KD. Lineage, fate, and fate potential of NG2-glia. Brain Res. 2015. 10.1016/j.brainres.2015.08.013 PMC4761528 26301825

[3] Hughes EG, Kang SH, Fukaya M, Bergles DE. Oligodendrocyte progenitors balance growth with self-repulsion to achieve homeostasis in the adult brain. Nat Neurosci. 2013;16(6):668–76. 10.1038/nn.3390 23624515 PMC3807738

[4] Lin SC, Bergles DE. Synaptic signaling between GABAergic interneurons and oligodendrocyte precursor cells in the hippocampus. Nat Neurosci. 2004;7(1):24–32. 14661022 10.1038/nn1162

[5] Bergles DE, Roberts JD, Somogyi P, Jahr CE. Glutamatergic synapses on oligodendrocyte precursor cells in the hippocampus. Nature. 2000;405(6783):187–91. 10821275 10.1038/35012083

[6] Bergles DE, Jabs R, Steinhauser C. Neuron-glia synapses in the brain. Brain Res Rev. 2010;63(1–2):130–7. 10.1016/j.brainresrev.2009.12.003 20018210 PMC2862892

[7] Yuan X, Chittajallu R, Belachew S, Anderson S, McBain CJ, Gallo V. Expression of the green fluorescent protein in the oligodendrocyte lineage: a transgenic mouse for developmental and physiological studies. J Neurosci Res. 2002;70(4):529–45. 12404507 10.1002/jnr.10368

[8] Zonouzi M, Scafidi J, Li P, McEllin B, Edwards J, Dupree JL, et al GABAergic regulation of cerebellar NG2 cell development is altered in perinatal white matter injury. Nat Neurosci. 2015;18(5):674–82. 10.1038/nn.3990 25821912 PMC4459267

[9] Kang SH, Fukaya M, Yang JK, Rothstein JD, Bergles DE. NG2+ CNS glial progenitors remain committed to the oligodendrocyte lineage in postnatal life and following neurodegeneration. Neuron. 2010;68(4):668–81. 10.1016/j.neuron.2010.09.009 21092857 PMC2989827

[10] Stallcup WB, Beasley L. Bipotential glial precursor cells of the optic nerve express the NG2 proteoglycan. J Neurosci. 1987;7(9):2737–44. 3305800 10.1523/JNEUROSCI.07-09-02737.1987 PMC6569154

[11] Belachew S, Chittajallu R, Aguirre AA, Yuan X, Kirby M, Anderson S, et al Postnatal NG2 proteoglycan-expressing progenitor cells are intrinsically multipotent and generate functional neurons. J Cell Biol. 2003;161(1):169–86. 12682089 10.1083/jcb.200210110 PMC2172886

[12] Kondo T, Raff M. Oligodendrocyte precursor cells reprogrammed to become multipotential CNS stem cells. Science. 2000;289(5485):1754–7. 10976069 10.1126/science.289.5485.1754

[13] Huang W, Zhao N, Bai X, Karram K, Trotter J, Goebbels S, et al Novel NG2-CreERT2 knock-in mice demonstrate heterogeneous differentiation potential of NG2 glia during development. Glia. 2014;62(6):896–913. 10.1002/glia.22648 24578301

[14] Komitova M, Serwanski DR, Lu QR, Nishiyama A. NG2 cells are not a major source of reactive astrocytes after neocortical stab wound injury. Glia. 2011;59(5):800–9. 10.1002/glia.21152 21351161 PMC3560299

[15] Tripathi RB, Rivers LE, Young KM, Jamen F, Richardson WD. NG2 glia generate new oligodendrocytes but few astrocytes in a murine experimental autoimmune encephalomyelitis model of demyelinating disease. J Neurosci. 2010;30(48):16383–90. 10.1523/JNEUROSCI.3411-10.2010 21123584 PMC3063541

[16] Tsoa RW, Coskun V, Ho CK, de Vellis J, Sun YE. Spatiotemporally different origins of NG2 progenitors produce cortical interneurons versus glia in the mammalian forebrain. Proc Natl Acad Sci U S A. 2014;111(20):7444–9. 10.1073/pnas.1400422111 24799701 PMC4034245

[17] Zhu X, Zuo H, Maher BJ, Serwanski DR, LoTurco JJ, Lu QR, et al Olig2-dependent developmental fate switch of NG2 cells. Development. 2012;139(13):2299–307. 10.1242/dev.078873 22627280 PMC3367441

[18] Simon C, Gotz M, Dimou L. Progenitors in the adult cerebral cortex: Cell cycle properties and regulation by physiological stimuli and injury. Glia. 2011;59(6):869–81. 10.1002/glia.21156 21446038

[19] Wilson H, Scolding N, Raine C. Co-expression of PDGF αreceptor and NG2 by oligodendrocyte precursor cells in human CNS and multiple sclerosis lesions. J Neuroimmunol. 2006;176:162–73. 16753227 10.1016/j.jneuroim.2006.04.014

[20] Baracskay KL, Kidd GJ, Miller RH, Trapp BD. NG2-positive cells generate A2B5-positive oligodendrocyte precursor cells. Glia. 2007;55(10):1001–10. 17503442 10.1002/glia.20519

[21] Bu J, Banki A, Wu Q, Nishiyama A. Increased NG2(+) glial cell proliferation and oligodendrocyte generation in the hypomyelinating mutant shiverer. Glia. 2004;48(1):51–63. 15326615 10.1002/glia.20055

[22] Trotter J, Karram K, Nishiyama A. NG2 cells: Properties, progeny and origin. Brain Res Rev. 2010;63(1–2):72–82. 10.1016/j.brainresrev.2009.12.006 20043946 PMC2862831

[23] Nishiyama A, Komitova M, Suzuki R, Zhu X. Polydendrocytes (NG2 cells): multifunctional cells with lineage plasticity. Nat Rev Neurosci. 2009;10(1):9–22. 10.1038/nrn2495 19096367

[24] Sakry D, Neitz A, Singh J, Frischknecht R, Marongiu D, Biname F, et al Oligodendrocyte precursor cells modulate the neuronal network by activity-dependent ectodomain cleavage of glial NG2. PLoS Biol. 2014;12(11):e1001993 10.1371/journal.pbio.1001993 25387269 PMC4227637

[25] Sakry D, Trotter J. The role of the NG2 proteoglycan in OPC and CNS network function. Brain Res. 2015. 10.1016/j.brainres.2015.06.003 26100334

[26] Sakry D, Yigit H, Dimou L, Trotter J. Oligodendrocyte precursor cells synthesize neuromodulatory factors. PLoS One. 2015;10(5):e0127222 10.1371/journal.pone.0127222 25966014 PMC4429067

[27] Kucharova K, Stallcup WB. The NG2 proteoglycan promotes oligodendrocyte progenitor proliferation and developmental myelination. Neuroscience. 2010;166(1):185–94. 10.1016/j.neuroscience.2009.12.014 20006679 PMC2847446

[28] Makagiansar IT, Williams S, Mustelin T, Stallcup WB. Differential phosphorylation of NG2 proteoglycan by ERK and PKCalpha helps balance cell proliferation and migration. J Cell Biol. 2007;178(1):155–65. 17591920 10.1083/jcb.200612084 PMC2064431

[29] Chekenya M, Rooprai HK, Davies D, Levine JM, Butt AM, Pilkington GJ. The NG2 chondroitin sulfate proteoglycan: role in malignant progression of human brain tumours. Int J Dev Neurosci. 1999;17(5–6):421–35. 10571405 10.1016/s0736-5748(99)00019-2

[30] Burg MA, Nishiyama A, Stallcup WB. A central segment of the NG2 proteoglycan is critical for the ability of glioma cells to bind and migrate toward type VI collagen. Exp Cell Res. 1997;235(1):254–64. 9281375 10.1006/excr.1997.3674

[31] Binamé F, Sakry D, Dimou L, Jolivel V, Trotter J. NG2 regulates directional migration of oligodendrocyte precursor cells via Rho GTPases and polarity complex proteins. J Neurosci. 2013;33(26):10858–74. 10.1523/JNEUROSCI.5010-12.2013 23804106 PMC6618486

[32] Binamé F. Transduction of extracellular cues into cell polarity: the role of the transmembrane proteoglycan NG2. Mol Neurobiol. 2014;50(2):482–93. 10.1007/s12035-013-8610-8 24390567

[33] Kucharova K, Chang Y, Boor A, Yong VW, Stallcup WB. Reduced inflammation accompanies diminished myelin damage and repair in the NG2 null mouse spinal cord. J Neuroinflammation. 2011;8:158 10.1186/1742-2094-8-158 22078261 PMC3229456

[34] Grako KA, Ochiya T, Barritt D, Nishiyama A, Stallcup WB. PDGF (alpha)-receptor is unresponsive to PDGF-AA in aortic smooth muscle cells from the NG2 knockout mouse. J Cell Sci. 1999;112 (Pt 6):905–15. 10036240 10.1242/jcs.112.6.905

[35] Moransard M, Dann A, Staszewski O, Fontana A, Prinz M, Suter T. NG2 expressed by macrophages and oligodendrocyte precursor cells is dispensable in experimental autoimmune encephalomyelitis. Brain. 2011;134(Pt 5):1315–30. 10.1093/brain/awr070 21596769

[36] Karram K, Chatterjee N, Trotter J. NG2-expressing cells in the nervous system: role of the proteoglycan in migration and glial-neuron interaction. J Anat. 2005;207(6):735–44. 16367801 10.1111/j.1469-7580.2005.00461.x PMC1571586

[37] Karram K, Goebbels S, Schwab M, Jennissen K, Seifert G, Steinhauser C, et al NG2-expressing cells in the nervous system revealed by the NG2-EYFP-knockin mouse. Genesis. 2008;46(12):743–57. 10.1002/dvg.20440 18924152

[38] Watkins TA, Emery B, Mulinyawe S, Barres BA. Distinct stages of myelination regulated by gamma-secretase and astrocytes in a rapidly myelinating CNS coculture system. Neuron. 2008;60(4):555–69. 10.1016/j.neuron.2008.09.011 19038214 PMC2650711

[39] Vana AC, Flint NC, Harwood NE, Le TQ, Fruttiger M, Armstrong RC. Platelet-derived growth factor promotes repair of chronically demyelinated white matter. J Neuropathol Exp Neurol. 2007;66(11):975–88. 17984680 10.1097/NEN.0b013e3181587d46 PMC2788485

[40] Preisner A, Albrecht S, Cui QL, Hucke S, Ghelman J, Hartmann C, et al Non-steroidal anti-inflammatory drug indometacin enhances endogenous remyelination. Acta Neuropathol. 2015;130(2):247–61. 10.1007/s00401-015-1426-z 25943886

[41] Ahlgren S, Li GL, Olsson Y. Accumulation of beta-amyloid precursor protein and ubiquitin in axons after spinal cord trauma in humans: immunohistochemical observations on autopsy material. Acta Neuropathol. 1996;92(1):49–55. 8811125 10.1007/s004010050488

[42] Nikic I, Merkler D, Sorbara C, Brinkoetter M, Kreutzfeldt M, Bareyre FM, et al A reversible form of axon damage in experimental autoimmune encephalomyelitis and multiple sclerosis. Nat Med. 2011;17(4):495–9. 10.1038/nm.2324 21441916

[43] de Castro F, Bribian A, Ortega MC. Regulation of oligodendrocyte precursor migration during development, in adulthood and in pathology. Cell Mol Life Sci. 2013;70(22):4355–68. 10.1007/s00018-013-1365-6 23689590 PMC11113994

[44] Simpson PB, Armstrong RC. Intracellular signals and cytoskeletal elements involved in oligodendrocyte progenitor migration. Glia. 1999;26(1):22–35. 10088669

[45] Milner R, Anderson HJ, Rippon RF, McKay JS, Franklin RJ, Marchionni MA, et al Contrasting effects of mitogenic growth factors on oligodendrocyte precursor cell migration. Glia. 1997;19(1):85–90. 8989571 10.1002/(sici)1098-1136(199701)19:1<85::aid-glia9>3.0.co;2-9

[46] Frost EE, Nielsen JA, Le TQ, Armstrong RC. PDGF and FGF2 regulate oligodendrocyte progenitor responses to demyelination. J Neurobiol. 2003;54(3):457–72. 12532397 10.1002/neu.10158 PMC7167702

[47] Fruttiger M, Karlsson L, Hall AC, Abramsson A, Calver AR, Bostrom H, et al Defective oligodendrocyte development and severe hypomyelination in PDGF-A knockout mice. Development. 1999;126(3):457–67. 9876175 10.1242/dev.126.3.457

[48] Paez PM, Fulton DJ, Spreuer V, Handley V, Campagnoni CW, Macklin WB, et al Golli myelin basic proteins regulate oligodendroglial progenitor cell migration through voltage-gated Ca2+ influx. J Neurosci. 2009;29(20):6663–76. 10.1523/JNEUROSCI.5806-08.2009 19458236 PMC2739626

[49] Miyamoto Y, Yamauchi J, Tanoue A. Cdk5 phosphorylation of WAVE2 regulates oligodendrocyte precursor cell migration through nonreceptor tyrosine kinase Fyn. J Neurosci. 2008;28(33):8326–37. 10.1523/JNEUROSCI.1482-08.2008 18701695 PMC6670578

[50] Frost EE, Zhou Z, Krasnesky K, Armstrong RC. Initiation of oligodendrocyte progenitor cell migration by a PDGF-A activated extracellular regulated kinase (ERK) signaling pathway. Neurochem Res. 2009;34(1):169–81. 10.1007/s11064-008-9748-z 18512152 PMC2678958

[51] Bansal R, Kumar M, Murray K, Morrison RS, Pfeiffer SE. Regulation of FGF receptors in the oligodendrocyte lineage. Mol Cell Neurosci. 1996;7(4):263–75. 8793862 10.1006/mcne.1996.0020

[52] Bribian A, Barallobre MJ, Soussi-Yanicostas N, de CF. Anosmin-1 modulates the FGF-2-dependent migration of oligodendrocyte precursors in the developing optic nerve. Mol Cell Neurosci. 2006;33(1):2–14. 16876430 10.1016/j.mcn.2006.05.009

[53] Xiao L, Hu C, Yang W, Guo D, Li C, Shen W, et al NMDA receptor couples Rac1-GEF Tiam1 to direct oligodendrocyte precursor cell migration. Glia. 2013;61(12):2078–99. 10.1002/glia.22578 . 24123220

[54] Passlick S, Trotter J, Seifert G, Steinhauser C, Jabs R. The NG2 Protein Is Not Required for Glutamatergic Neuron-NG2 Cell Synaptic Signaling. Cereb Cortex. 2016;26(1):51–7. 10.1093/cercor/bhu171 . 25100858

[55] Kadoya K, Fukushi J, Matsumoto Y, Yamaguchi Y, Stallcup WB. NG2 proteoglycan expression in mouse skin: altered postnatal skin development in the NG2 null mouse. J Histochem Cytochem. 2008;56(3):295–303. 18040080 10.1369/jhc.7A7349.2007 PMC2324176

[56] Burg MA, Grako KA, Stallcup WB. Expression of the NG2 proteoglycan enhances the growth and metastatic properties of melanoma cells. J Cell Physiol. 1998;177(2):299–312. 9766527 10.1002/(SICI)1097-4652(199811)177:2<299::AID-JCP12>3.0.CO;2-5

[57] Sugiarto S, Persson AI, Munoz EG, Waldhuber M, Lamagna C, Andor N, et al Asymmetry-defective oligodendrocyte progenitors are glioma precursors. Cancer Cell. 2011;20(3):328–40. 10.1016/j.ccr.2011.08.011 21907924 PMC3297490

[58] Huang C, Sakry D, Menzel L, Dangel L, Sebastiani A, Kramer T, et al Lack of NG2 exacerbates neurological outcome and modulates glial responses after traumatic brain injury. Glia. 2015. 10.1002/glia.22944 26638112

[59] Remington LT, Babcock AA, Zehntner SP, Owens T. Microglial recruitment, activation, and proliferation in response to primary demyelination. Am J Pathol. 2007;170(5):1713–24. 17456776 10.2353/ajpath.2007.060783 PMC1854965

[60] Boretius S, Escher A, Dallenga T, Wrzos C, Tammer R, Bruck W, et al Assessment of lesion pathology in a new animal model of MS by multiparametric MRI and DTI. Neuroimage. 2012;59(3):2678–88. 10.1016/j.neuroimage.2011.08.051 21914485

[61] Matsushima GK, Morell P. The neurotoxicant, cuprizone, as a model to study demyelination and remyelination in the central nervous system. Brain Pathol. 2001;11(1):107–16. 11145196 10.1111/j.1750-3639.2001.tb00385.x PMC8098267

[62] Gensert JM, Goldman JE. Endogenous progenitors remyelinate demyelinated axons in the adult CNS. Neuron. 1997;19:197–203. 9247275 10.1016/s0896-6273(00)80359-1

[63] Keirstead HS, Blakemore WF. Identification of post-mitotic oligodendrocytes incapable of remyelination within the demyelinated adult spinal cord. J Neuropathol Exp Neurol. 1997;56:1191–201. 9370229 10.1097/00005072-199711000-00003

[64] Crawford AH, Tripathi RB, Foerster S, McKenzie I, Kougioumtzidou E, Grist M, et al Pre-Existing Mature Oligodendrocytes Do Not Contribute to Remyelination following Toxin-Induced Spinal Cord Demyelination. Am J Pathol. 2016;186(3):511–6. 10.1016/j.ajpath.2015.11.005 26773350 PMC4816704

