# Correction: Recovery from Toxic-Induced Demyelination Does Not Require the NG2 Proteoglycan

**DOI:** 10.1371/journal.pone.0194322

**Published:** 2018-03-08

**Authors:** The PLOS ONE Journal Staff

The Abstract section is missing. The publisher apologizes for the error. The Abstract can be viewed here:

## Abstract

The expression of the proteoglycan NG2 is characteristic for oligodendroglial progenitor cells (OPC). The protein has been reported to influence OPC behavior in different pathological models. Here we examined the function of NG2 in remyelination ensuing after cuprizone feeding of mice to induce demyelination. OPC isolated from NG2-deficient mice exhibited an increased chemotaxis compared to WT OPC, but no differences in proliferation, differentiation or *in vivo* remyelination were observed. Our data suggest that NG2 modulates oligodendroglial migration but is dispensable for successful remyelination after cuprizone-induced demyelination.
